# Gene expression profile of placentomes and clinical parameters in the cows with retained placenta

**DOI:** 10.1186/s12864-022-08989-5

**Published:** 2022-11-21

**Authors:** Mehdi Moradi, Mahdi Zhandi, Mohsen Sharafi, Arvand Akbari, Mohammad Jafari Atrabi, Mehdi Totonchi

**Affiliations:** 1 Department of Animal Science, College of Agriculture and Natural Resources, University of Tehran, Karaj, Iran; 2 Department of Animal Science, Faculty of Agriculture, Tarbiat Modares University, Tehran, Iran; 3 Department of Embryology, Reproduction Biomedicine Research Center, Royan Institute for Reproductive Biomedicine, ACER, Tehran, Iran; 4 Department of Genetics, Reproductive Biomedicine Research Center, Royan Institute for Reproductive Biomedicine, ACECR, Tehran, Iran; 5 Institute of Pharmacology and Toxicology, University Medical Center, Georg August University, Göttingen, Germany

**Keywords:** Retained placenta, Cattle, RNA-seq, Placentome

## Abstract

**Background:**

Retained placenta (RP) is a prevalent disorder in cattle with many health-related and economic costs for the farm owners. Its etiology has not been clarified yet and there is no definite therapy for this disorder. In this study we conducted RNA-seq, hematologic and histologic experiments to survey the causes of RP development.

**Methods:**

Blood samples were collected from 4 RP and 3 healthy cows during periparturtion period for hematological assessments followed by placentome sampling within 30 min after parturition. Cows were grouped as RP and control in case the placenta was retained or otherwise expelled, respectively. Total RNA was extracted from placentome samples followed by RNA-sequencing.

**Results:**

We showed 240 differentially expressed genes (DEGs) between the RP and control groups. Enrichment analyzes indicated immune system and lipid metabolism as prominent over- and under-represented pathways in RP cows, respectively. Hormonal assessments showed that estradiol-17β (E2) was lower and cortisol tended to be higher in RP cows compared to controls at the day of parturition. Furthermore, histologic experiment showed that villi-crypt junctions remain tighter in RP cows compared to controls and the crypts layer seemed thicker in the placentome of RP cows. Complete blood cell (CBC) parameters were not significantly different between the two groups.

**Conclusion:**

Overall, DEGs derived from expression profiling and these genes contributed to enrichment of immune and lipid metabolism pathways. We suggested that E2 could be involved in development of RP and the concentrations of P4 and CBC counts periparturition might not be a determining factor.

**Supplementary Information:**

The online version contains supplementary material available at 10.1186/s12864-022-08989-5.

## Background

Retained placenta (RP) is a prevalent disorder in cattle with many negative health- and reproduction-related implications for the cow and economic losses for the farms [1–3]. The incidence of RP can range from 1.3 to 39.2% in different herds [4]. Retained placenta correlates with reduced uterine chemotaxis and immunity and potentially affects uterine involution [2, 5–7]. This disorder is also associated with decreased milk yield, delayed return to heat after calving, increased days open and number of inseminations and reduced conception rate [2, 3]. The central sites of problem in the occurrence of RP are placentomes. Placentomes in cattle are the sites of fetomaternal interactions and substance exchanges. Fetal villi are fitted into maternal crypts through various adhering links that maintain these structures closely attached to each other throughout the gestation [2, 8]. By approaching parturition, placentome maturation and degradation of extracellular matrix (ECM) between the fetal and maternal sides lead to loosening of the existing links. This placentome maturation is partly under hormonal influence of estrogen and progesterone [9]. Upon parturition, a culmination of ECM degradation, apoptotic events and morphologic changes help in separation and expulsion of placenta from the maternal caruncles [10, 11]. Retained placenta is defined as a condition, in which fetal membranes are not expelled within 12 h after parturition [2]. Despite numerous studies, the etiology of RP is not clear yet and there is no definite therapy for this disorder. The inclusion of immune system, deficiency in degradation of ECM and hormonal imbalances have been described as the probable etiologies of RP and some molecules such as major histocompatibility complex (MHC) class I, matrix metalloproteinases (MMPs) and Prostaglandin F2α have been attributed to this disorder [12]. Defective immunogenic signaling between fetal and maternal tissue in placentomes is a major contributor to placental retention [13]. Decreased activity of macrophages and neutrophils is associated with increased incidence of RP [5, 14]. Some hematologic alternations such as neutrophil decline and increased red blood cell count in the blood of cows with retained placenta compared to healthy ones have also been reported [15]. Bacterial invasions due to defective immune defense are also mentioned as one of the etiologies of RP [16]. Various cell types including leukocytes have been claimed to release proteolytic enzymes such as MMPs around the time of parturition and it has been shown that the release and activity of collagenases could be affected by various upstream factors such as progesterone, serotonin, relaxin and cytokines [10, 12, 17, 18]. In addition, a network of metabolic disturbances, hormonal imbalances and immune dysfunctions has been proposed as the causes of RP [19–24]. Nutrition and metabolic status of cows could involve in retention of placenta [25] and RP could be higher in the cows with intense fat metabolism [26]. The link between the level of lipid compounds and incidence of RP has been reported by various studies and it seems that the levels of cholesterol, HDL and fatty acids need to be monitored in transition period [27–31]. Elevated lactate is another risk factor for RP in cattle. In one study, lactate remained elevated in RP cows from 8 weeks before parturition to the week of parturition and even to 4 weeks after parturition [22].

Rapid hormonal changes in plasma concurrent with reduced luteal function and pre-partum stimulation of placental P450c17 activity are among the fundamental physiologic events which occur during periparturition period and affect normal calving and expulsion of fetal membranes [10, 32, 33]. Dysfunction of aromatase activity has been attributed to insufficient estrogen production and difficulties in expulsion of fetal membranes [10]. In this regards, downregulation of CYP19, which is involved in conversion of progesterone to estrogen has been mentioned as a potential contributor to development of RP [34]. Dysregulation of cortisol, progesterone and estradiol-17β are always receiving attention when considering the hormonal differences between cows with RP and healthy cows. However, the link between the concentration of these hormones and development of RP is somewhat controversial. While in some studies, periparturition plasma concentrations of these hormones have been different between RP and healthy cows, other studies have not seen a similar meaningful difference [9, 23, 35, 36]. A whole transcriptome study by microarray determined the gene sets that were differentially expressed in placentomes obtained from the cows with early induced parturition compared to placentomes harvested after normal calving [11]. Genes associated with immune system and apoptosis and also MMPs as extracellular matrix remodeling factors were enriched in this study. However, this study has not directly compared the whole transcriptome of RP and healthy cows. In order to shed more light on the mechanisms of RP development, we performed clinical investigation on the cows with or without RP, followed by a gene expression profiling on the placentomes collected from the cows.

## Materials and methods

### Animals and sampling

Holstein cows included in this study were held in the same barn in the last month before parturition and were fed with the same diet. Blood samples were collected from the cows that had signs of parturition in the following days twice a day at 08:00 AM and 06:00 PM using 10 ml EDTA-tubes. We did not use heifers in this study. One milliliter of each blood sample was used for CBC assessment. Plasma was separated from the rest of the blood sample by centrifugation at 900 × g for 15 min. Plasma was then maintained at -80^◦^C for hormonal assessments. All the cows delivered a calf with normal health status. Within 30 min after parturition, placentomes were collected from the cows according to the method described previously [37]. In brief, a 0.3 mm steel acoustic guitar string and a brass tubing were used as a placentome collection device. A loop was made on one end of the string and the other end was passed through three holes drilled on the shaft of the brass tubing as a handling object. The anus and vaginal area of the cow was cleaned and disinfected using warm water and povidone-iodine 10%, respectively. Then, the hand of the operator was covered with an arm length veterinary glove and the collection loop was passed through the vagina reaching the placentomes in the uterus. The loop was positioned at the base of the caruncle and the placentome was separated by pulling the handle outward and closing of the loop. Three placentomes from each cow were collected, washed with normal saline and placed in phosphate buffer saline containing 10 mg streptomycin and 1 mg penicillin per 100 ml, immediately [38]. The fetal-maternal junction was retained and not separated. Biopsies were then taken from the placentomes by systematic random sampling [39] according to the previous study [11]. In brief, placentomes were sliced vertically from the fetal to the maternal side by 0.5 cm cuts. The slices were placed under a 1 × 1 cm grid and random biopsy cuboids were taken from the fetal-maternal junction site of the slices. The cuboids were divided into two equal smaller cuts. One cuboid was placed in liquid nitrogen for RNA-Seq assay and real-time PCR, while the other one was placed in 10% neutral buffered formalin for histologic experiments. The other sampled cuboids from the slices were cut and stored in the same way and were pooled with the previous ones. The tissue cuboids fixed in formalin were processed routinely and embedded in paraffin.

The cows that did not expel their placenta until 24 h after parturition were considered as RP and the cows that expelled their placenta within 24 h after parturition were considered as normal. However, all the normal cows in this study expelled their placenta within 12 h after parturition and none of the RP cows expelled its placenta even after 24 h postparturition and they received veterinary therapies thereafter.

Sample collection was continued until four cows showed placental retention and eight cows had normal placental expulsion. Then, the four RP cows (RP group) and three randomly selected healthy cows (Control group) were used for further experiments including RNA-sequencing. Information about the cows included in this study is shown in Table 1. Mean values between the two groups were compared using student’s T-test.

**Table 1:** Information about the cows used in this study

**Subject**	**Parity**	**Calf gender**	**Milk yield in previous lactation (kg)**	**Length of last gestation (days)**	**Age at the time of last parturition (days)**	**Age at first pregnancy (days)**	**Mean interval between each parity after first pregnancy (days)**	**Length of last days open**
Control 1	8th	Female	8411	285	4315	701	452	251
Control 2	6th	Male	8690	281	2667	457	368	125
Control 3	7th	Male	10,223	282	3041	440	372	85
RP 1	3rd	Female	11,480	287	2021	594	476	240
RP 2	4th	Male	12,492	278	1858	423	359	91
RP 3	5th	Male	8796	284	2470	541	386	177
RP 4	6th	Female	7930	281	2738	452	381	103
Mean of Controls (± Sd)	7 ± 1		9108 ± 975	282.6 ± 2.1	3341 ± 864	533 ± 146	397 ± 47	154 ± 87
Mean of RPs (± Sd)	4.5 ± 1.3^a^		10,174 ± 2161	282.5 ± 3.9	2272 ± 404	503 ± 79	401 ± 52	153 ± 69

^a^Significantly different means between the groups (*P* < 0.05)

### Assessment of blood hormones and hematological parameters

Plasma concentrations of estradiol-17β (E2), progesterone (P4) and cortisol were assessed using a commercial ELISA kit (Catalog No. DEH3355, DE1561 and DEH3388, respectively; Demeditec, GmbH, Germany), according to the manufacturer’s protocol. Assay ranges demonstrated by the manufacturer for E2, P4 and cortisol were 10.6 pg/mL – 2000 pg/mL, 0.140 ng/mL – 40 ng/mL and 10 ng/mL – 800 ng/mL, respectively. Intra-assay coefficient of variation calculated by our lab for all the three hormonal assessments was less than 10%.

Hematological parameters (red blood cell count, white blood cell count, mean corpuscular hemoglobin, mean corpuscular hemoglobin concentration, mean corpuscular volume, platelet count, packed cell volume and hemoglobin concentration) were also measured by a Hematology Analyzer (Seac-Radim Hemat 8, Italy). The percentage of each leukocyte cell population on blood smears was measured as well, using a light microscope immediately after samples were delivered to laboratory. The mean of the two daily blood assessments was calculated and presented as the value for each periparturtion day to minimize the effect of daily alternations in the parameters.

RStudio® was used for statistical analysis of the hormonal and hematological parameters. Data normality was checked using Shapiro Wilk’s test function. Repeated measures ANOVA and pairwise between-subject comparisons in each time point were also conducted using nlme (version 3.1.152) and emmeans (version 1.6.0, method: tukey adjusted *p* value) packages, respectively. *P*-values less than 0.05 considered as statistically significant.

### Hematoxylin-eosin staining

Placentome tissue sections were prepared from paraffin-embedded tissues using a microtome. The sections were deparaffinized by xylene and hydrated by 100%, 96% and 70% ethanol and subsequently by distilled water followed by hematoxylin and eosin staining and dehydration by 70%, 96% and 100% ethanol and xylene, respectively. Finally, the sections were mounted by Entellan™ rapid mounting medium (Catalog No. 1.07961; Merck, Germany) and investigated under a light microscope.

## Gene expression profiling

### RNA extraction and sequencing

Total RNA was extracted from placentome biopsies using TRIzol™ (Catalog No. 15,596,018, USA) reagent and following routine extraction procedures. Total extracted RNA was then treated with DNase I (Catalog No. 18,068,015, Invitrogen™, USA) and finally, the quantity of total RNA was measured by NanoDrop™ (Thermo Fisher Scientific, USA) and its quality was assessed by BioAnalyzer 2100 (Agilent Technologies, USA). Total RNAs from all samples had RIN score of 7 or higher. RNA samples were shipped to Genotypic Institute in India for RNA-sequencing. Illumina’s protocols were used for poly(A) selection and paired-end sequencing was performed using Illumina HiSeq X ten platform. Average read lengths was set to 150 bp.

For RT-qPCR experiments, a remaining part of the extracted total RNA (equalized concentration for all samples) was reverse transcribed using ExcelRT™ reverse transcription kit (Catalog No. RP1300, SMOBIO, Taiwan), according to the manufacturer’s protocol. To perform qPCR assessment, duplicate reactions were prepared for each sample using RealQ Plus 2x Master Mix Green (Catalog No. A323402, Ampliqon, Denmark), forward and reverse primers (0.2 µM each) and cDNA (25 ng), adjusted to total volume of 10 µL using molecular grade H_2_O. Amplification cycles continued for 40 cycles using an ABI stepone-plus™ real-time PCR instrument. The amplification efficiency and relative expression (RPs compared to controls) for each gene were calculated using the method described previously [40]. The sequences of primers used in this study are provided in Table S1 (see Additional file 1).

### Bioinformatic analysis of sequencing data

The quality of raw data was assessed by Fastqc [41] and the reads were trimmed using Trimmomatic [42]. The trimmed reads were aligned to the reference genome (ARS-UCD1.2) by HISAT2 [43] and the number of reads assigned to each gene was determined using featureCounts [44]. Differentially expressed genes between the RP and control groups were determined using EdgeR [45]. To identify DEGs, a cutoff (median log CPM > -2.2) was initially set for the genes read counts to exclude the genes with very low read counts throughout the samples. Then, effective library sizes were calculated by trimmed mean of M-values (TMM). Dispersions estimated via quantile-adjusted conditional maximum likelihood (qCML) and DEGs were determined by the exact test. Genes with FDR < 0.05 and fold change > 1.5 or < -1.5 were considered as significantly DEGs.

Gene ontology analysis was done by over-representation analysis (ORA) on upregulated and downregulated DEGs, separately, via Webgestalt online tool [46]. The STRING online tool (https://string-db.org) was used to investigate the interaction network between the upregulated and downregulated genes [47]. Protein-protein interaction (PPI) option was used to retrieve the potential interactions between the proteins. Medium confidence (0.4) was set for the interaction scores. To further explore how E2 could hypothetically affect the drawn PPI networks from the up- and down regulated genes, we imported annotated nuclear E2 receptors (ESR1 and ESR2) to the above networks as well. The retrieved network from STRING (excluding ESR1 and ESR2) was then transferred to Cytoscape software (v3.8.2) to survey the functional enrichment of the interacting proteins [48].

## Results

### Clinical data of the cows

**Fig. 1:**
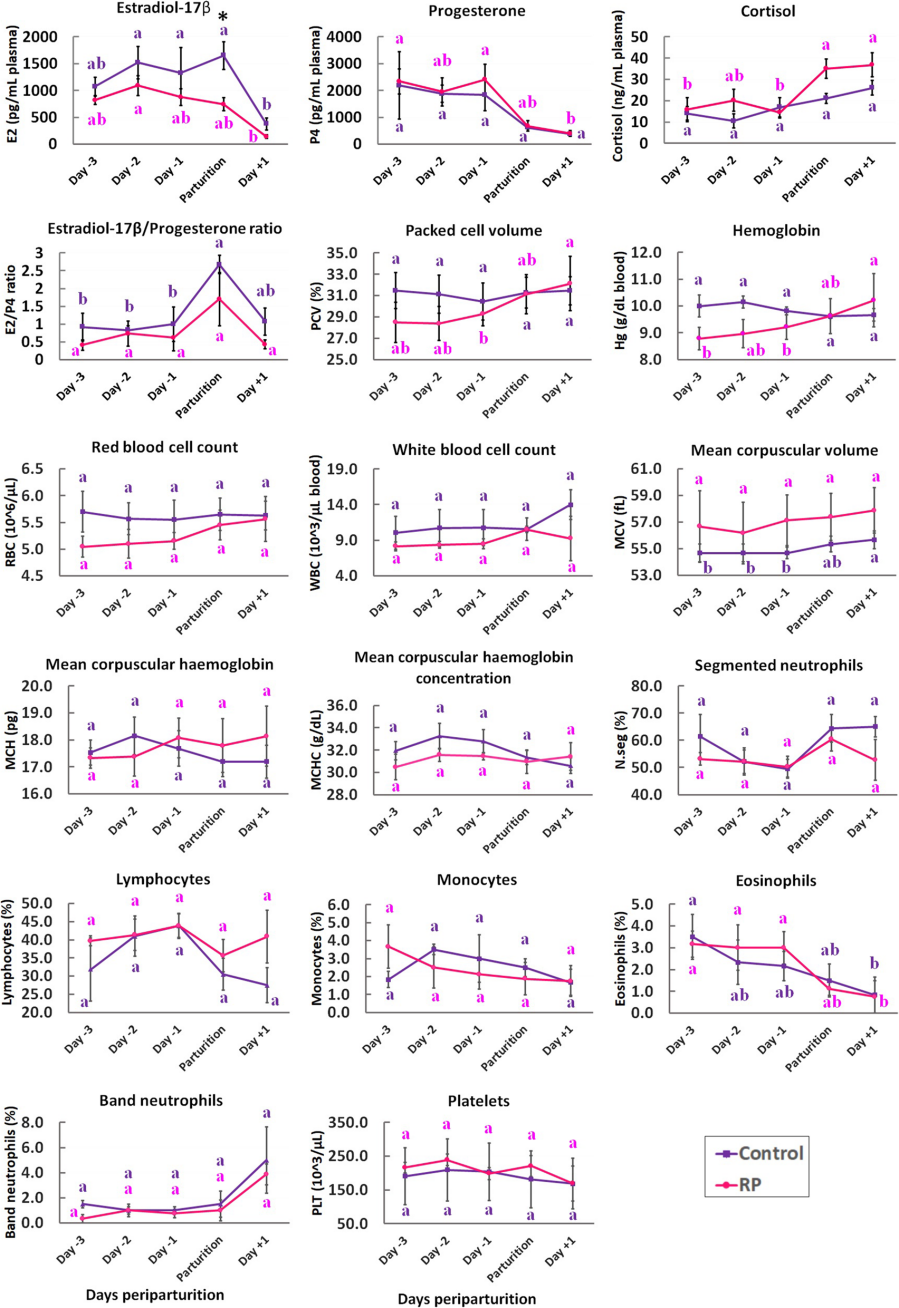
Concentration of hormones and various blood parameters in RP and control groups during periparturition days. The level of E2 at the parturition day was significantly different between the RP and control groups (*P* = 0.025; showed by asterisk). Other parameters were not significantly different between the groups (*P* > 0.05). Letters with corresponding colors have been used to indicate within group significant changes between different periparturition time points. Different letters indicate significant differences (*P* < 0.05). Each data point is shown as Mean ± SE

Plasma concentration of E2, P4, cortisol and the E2/P4 ratio and various blood cell parameters in RP and control groups in periparturition days were assessed. The time × group interaction was not significant (*P* > 0.05) for all the above variables. The concentration of E2 was significantly lower (*P* = 0.025) in RP group, compared to controls at the day of parturition (Fig. 1). Its concentration was also higher in controls than RPs in all the days periparturtion, however, the differences were not statistically significant. At the day + 1, the concentration of E2 dropped to low levels (lower than 400 pg/mL plasma) in the both groups and its decrease was significant within each group compared to parturition or preparturition days (Fig. 1).

No significant difference in P4 concentrations was observed between RP and control groups. Similar to E2, the concentration of P4 decreased to low levels at the day + 1 and its decrease was significant compared to the days − 1 and day − 3 within the RP group (Fig. 1). Although E2/P4 ratio changed similarly in the RP and control groups, the temporary elevation of this parameter in the day of parturition was only significant (*P* < 0.05) within the control group compared to preparturition days (Fig. 1). Cortisol concentration tended to be higher (*P* = 0.068) in RPs than controls in the day of parturition. It also showed significantly higher concentration in RP (*P* < 0.05) and not control group in the day of parturition and day + 1, compared to days − 1 and − 3 (Fig. 1).

None of the blood cell parameters were significantly different between the RP and control groups. However, packed cell volume, hemoglobin, mean corpuscular volume and eosinophils changed significantly within RP, control or both groups at different days periparturition (Fig. 1). In RPs, hemoglobin showed an increasing pattern during the days preparturition to postparturition and it reached to significantly higher level at day + 1 compared to days − 1 and − 3. In controls, this parameter was almost constant in all the days periparturition (Fig. 1). Packed cell volume experienced a similar trend to hemoglobin in the both groups and within RP group, its level was significantly different between days + 1 and − 1 (Fig. 1). Mean corpuscular volume was significantly higher in the day + 1 compared to the days preparturition within the control group (Fig. 1). Eosinophils showed a decreasing pattern in the both groups and its decrease was significant between the day + 1 and the days preparturition in RP and the day − 3 in control group (Fig. 1). Red and white blood cell count had an almost constant level in the both groups during periparturtion days. However, white blood cell count was non-significantly higher in control group than RPs in day + 1 (*P* > 0.05). Segmented neutrophils and lymphocytes were lower and higher, respectively, by about 15% in RPs than controls in day + 1. However, the differences were not significant (*P* > 0.05). Mean corpuscular hemoglobin, mean corpuscular hemoglobin concentration, monocytes, band neutrophils and platelets were not significantly different within the groups at different time points.

**Fig. 2:**
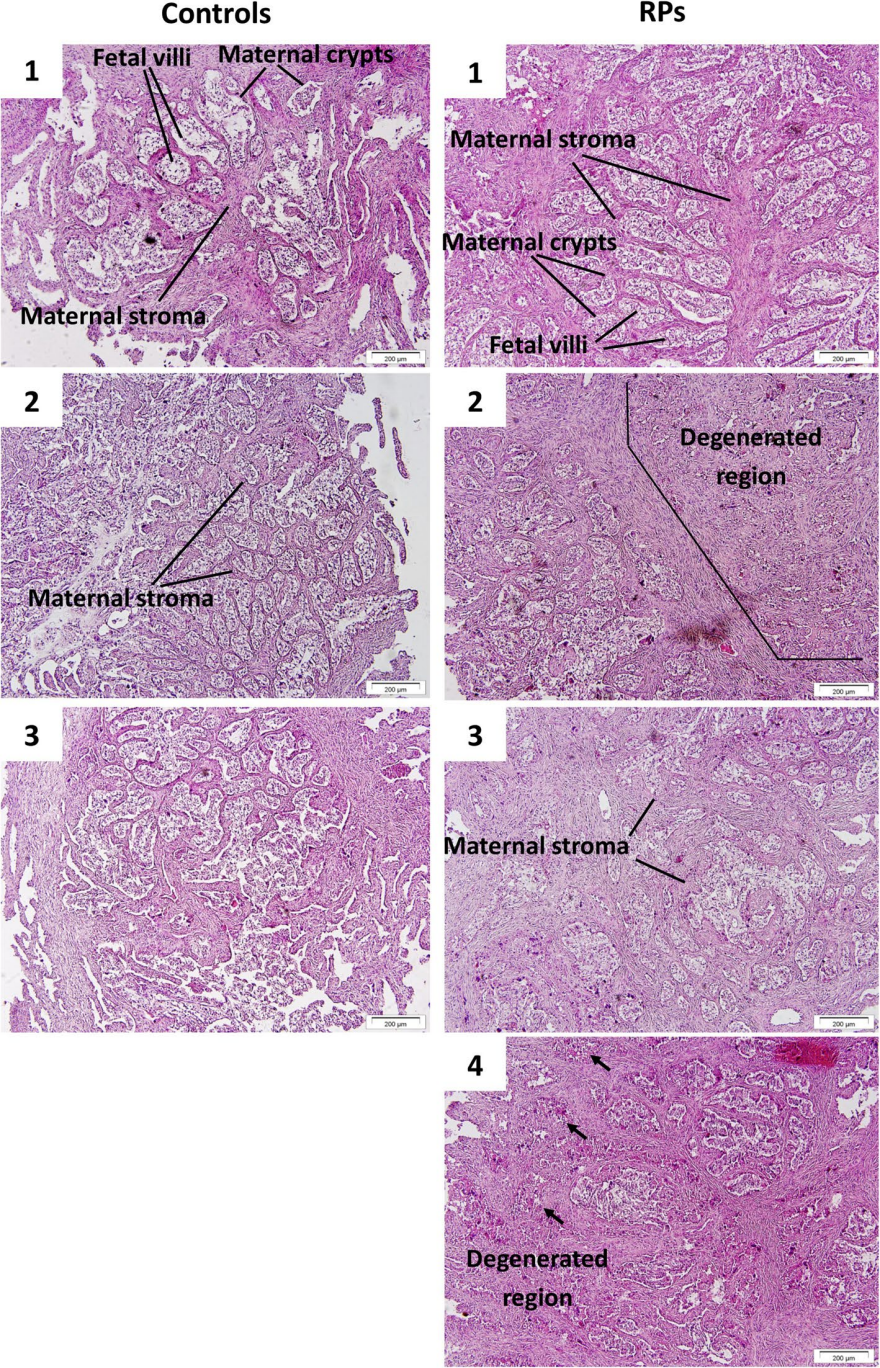
Transverse sections of the cows’ placentomes. The villi-crypts intersections are looser in the control cows compared to the RP ones which are seen as wider white spaces between fetal villi and maternal crypts. The maternal stroma seems to be thicker in some regions in RP cows. Some collapsed or degenerated structures which probably used to be intact villi-crypts are also seen in some regions of the placentome sections of RP cows. Arrow heads specifically show the collapsed villi-crypts which may be due to the elimination of the existing spaces between the fetal villi and maternal crypts. The cows’ number are corresponding to the cows used for RNA-sequencing

The fetal villi and maternal crypts of the placentomes were loose and somewhat detached in healthy cows. On the other hand, in RP cows the attachments were more firm and sustained. Some collapsed structures were seen in RP placentomes which might be associated with development of RP due to impaired detachment (Fig. 2).

### **Overview of the RNA-Seq data and gene expression profile of the RP and control cows**

#### Accession number

All RNA-seq raw data and raw read count matrix have been deposited into the Gene Expression Omnibus (GEO) under accession number GEO: GSE194033.

#### RNA-seq data analysis

Information about the total reads produced, alignment rates and total counted reads per sample is available in Table S2 (see Additional file 1). After counting the reads by featureCounts, an approximate average of 22,280,000 reads were mapped to each sample and an average of 807 reads were mapped to each gene in the samples before cleaning the genes with low read count. After excluding the genes with a median count per million (CPM) of the reads lower than − 2.2, 16,723 genes (out of 27,607 total genes) were retained for differential expression analyzes and an average of 1332 reads were mapped to each gene throughout the samples. Also, an approximate average of 22,275,000 reads for each sample remained after filtering the low read counts.

**Fig. 3:**
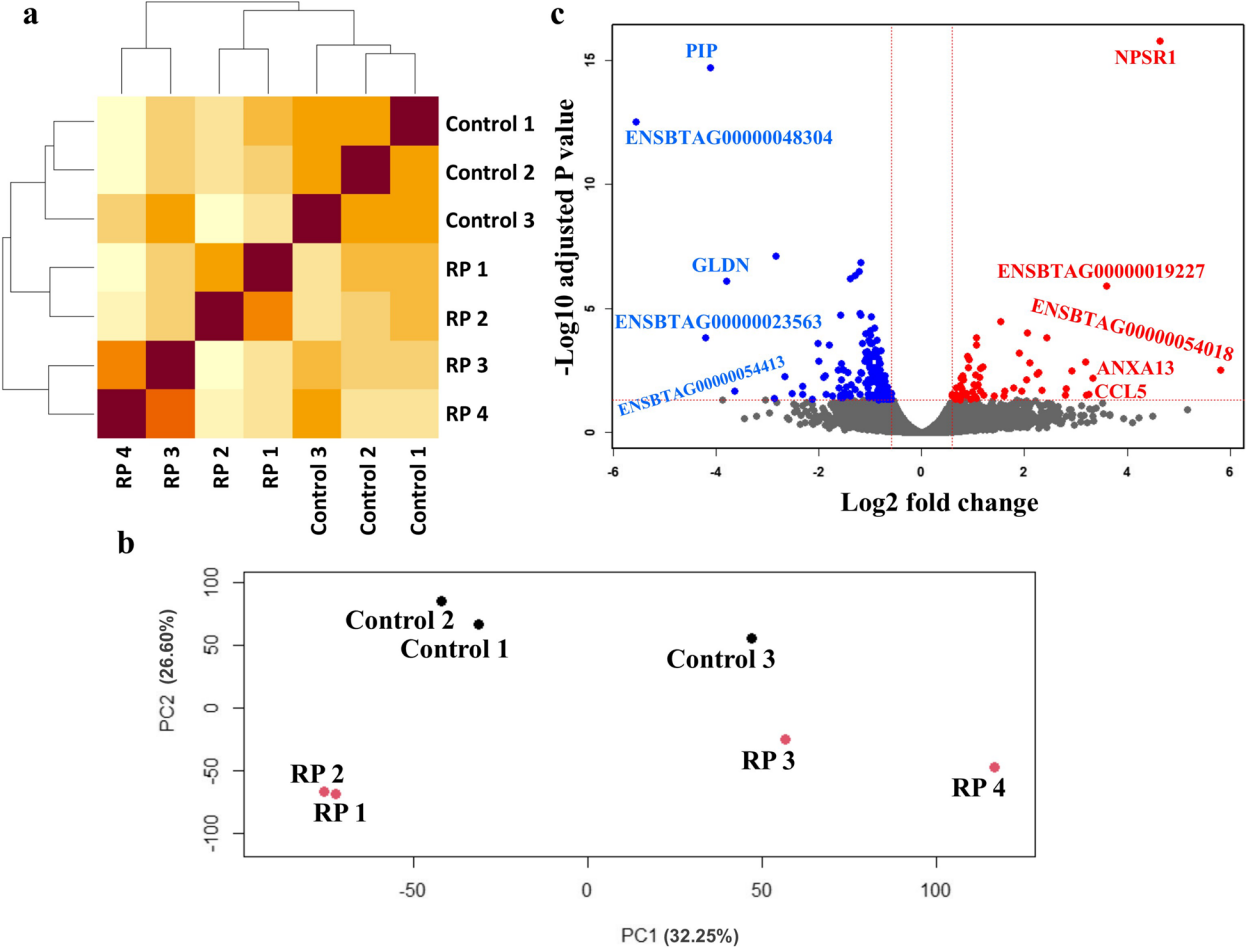
**a** Correlation heatmap shows the relationship between different samples. **b** PCA scatter plot shows the level of variations between different samples. Regarding the proximity between samples, a control/RP1-RP2/RP3-RP4 clustering could be deduced. Note that in the RP group, RP1 and RP4 had female and RP2 and RP3 had male calves. Also, in the control group, Control 1 had female and Control 2 and Control 3 had male calves. The percentage of variations obtained by the principal components is specified on each axis. **c** Volcano plot shows the distribution of the DEGs. Genes with –log10 FDR > 1.3 and log2 fold change > 0.59 or < -0.59 considered as significant (shown with red and blue circles, respectively). Top 5 genes (upregulated or downregulated) with highest relative expression (RP vs. control) have been specified with their gene symbol (or gene ID if the gene is not yet annotated)

Samples correlation heatmap and PCA plot are shown in Fig. 3a and b. These plots visualize gene expression relationship between the samples. As indicated, control samples have a higher correlation with each other. RP1 shows a higher correlation with RP2. Also, RP3 shows a higher correlation with RP4. However, RP1 and RP2 seem to have less pronounced correlation with RP3 and RP4.

EdgeR yielded a total of 240 genes with significant differential expression out of which, 64 genes upregulated while 176 genes downregulated in the RP group in comparison with control group (Table S3 (Additional file 1)). The volcano plot depicts the distribution of analyzed genes by their log2 fold change (x axis) and –log10 adjusted *P* value (y axis) (Fig. 3c).

#### Functional annotation of gene sets

Upregulated genes did not show enriched biological process with FDR < 0.05. However, top 10 enriched process option was used to obtain the biological processes that had highest over-representation. “Mature B cell differentiation”, “Positive regulation of lymphocyte migration” and “T-helper 1 type immune response” were the pathways with highest enrichment ratio. The over-represented processes and their corresponding genes are shown in Table 2. Top 10 biological processes obtained through ORA on downregulated genes enriched “fatty acid metabolic process”, “monocarboxylic acid metabolic process”, “oxoacid metabolic process”, “organic acid metabolic process” with FDR < 0.05. Complete list of the top 10 under-represented pathways and their corresponding genes are shown in Table 3.

**Table 2:** Top 10 over-represented biological processes enriched by up-regulated genes. The genes contributed to enrichment of each biological process are specified by italic characters

**Enriched biological processes and their associated genes**	**Enrichment ratio**	***P *** **value**
Mature B cell differentiation *CMTM7, LGALS1*	52.878	0.00059822
Positive regulation of lymphocyte migration *CXCL14, ITGB3*	26.439	0.0025034
T-helper 1 type immune response *IL27RA, RELB*	24.884	0.0028288
Positive regulation of cell-matrix adhesion *ITGB3, NINJ1*	23.502	0.0031729
Positive regulation of cell-substrate adhesion *ARL2, ITGB3, NINJ1*	15.108	0.00098375
Positive regulation of leukocyte migration *CXCL14, ITGB3, THBS4*	13.220	0.0014535
Positive regulation of peptidyl-tyrosine phosphorylation *THBS4, UNC119, HGF*	10.755	0.0026362
Regulation of peptidyl-tyrosine phosphorylation *ITGB3, THBS4, UNC119, HGF*	9.4006	0.00080833
Peptidyl-tyrosine phosphorylation *ITGB3, THBS4, UNC119, HGF*	6.1756	0.0037929
Peptidyl-tyrosine modification *ITGB3, THBS4, UNC119, HGF*	6.0432	0.0040986

No bolded biological process shows that none of the processes had FDR < 0.05

**Table 3:** Top 10 over-represented biological processes of down-regulated genes. The genes contributed to enrichment of each biological process are specified by italic characters

**Enriched biological processes and their associated genes**	**Enrichment ratio**	***P *** **value**
Glucosylceramide metabolic process *FA2H, UGCG*	60.2	0.000403
Response to platelet aggregation inhibitor *GNAI1, FDX1*	40.133	0.000997
**Fatty acid metabolic process** *ACSL1, SLC27A6, PLA2G15, FA2H, FASN, PDK1, CPT1A, HACD2, ACOX1*	**6.7304**	**6.29E-06**
**Monocarboxylic acid metabolic process** *CYP1A2, ACSL1, SLC27A6, ATP8B1, PLA2G15, FA2H, FASN, PDK1, CPT1A, HACD2, ACOX1*	**5.1734**	**6.92E-06**
**Oxoacid metabolic process** *CYP1A2, GLCE, ACSL1, SLC27A6, ATP8B1, OAT, PLA2G15, AASS, FA2H, BCAT1, FASN, PDK1, CPT1A, HACD2, ACOX1*	**3.6857**	**8.1E-06**
**Organic acid metabolic process** *CYP1A2, GLCE, ACSL1, SLC27A6, ATP8B1, OAT, PLA2G15, AASS, FA2H, BCAT1, FASN, PDK1, CPT1A, HACD2, ACOX1*	**3.612**	**1.03E-05**
**Carboxylic acid metabolic process** *CYP1A2, ACSL1, SLC27A6, ATP8B1, OAT, PLA2G15, AASS, FA2H, BCAT1, FASN, PDK1, CPT1A, HACD2, ACOX1*	**3.6017**	**2.23E-05**
Cellular lipid metabolic process *CYP1A2, ACSL1, SLC27A6, PLA2G15, FA2H, FASN, UGCG, PDK1, PLD1, CPT1A, HACD2, ACOX1*	3.1965	0.000291
Lipid metabolic process *CYP1A2, ACSL1, SLC27A6, ATP8B1, PLA2G15, FDX1, FA2H, FASN, UGCG, PDK1, PLD1, CPT1A, HACD2, ACOX1*	2.8473	0.000283
Small molecule metabolic process *CYP1A2, GLCE, AHCYL2, ACSL1, SLC27A6, ATP8B1, OAT, PLA2G15, ACPP, FDX1, AASS, FA2H, BCAT1, FASN, PDK1, PDXK, CPT1A, EPM2AIP1, HACD2, ACOX1*	2.5536	4.69E-05

Biological processes with FDR< 0.05 are shown by bold characters

Top 10 upregulated and downregulated biological processes retrieved through gene set enrichment analysis (GSEA) as well (Fig. S1 and Tables S4 and S5 (see Additional file 1)). The main part of upregulated pathways enriched in this analysis were related to immune system. On the other hand, the main part of downregulated pathways was related to lipid metabolism.

### PPI network analysis

**Fig. 4:**
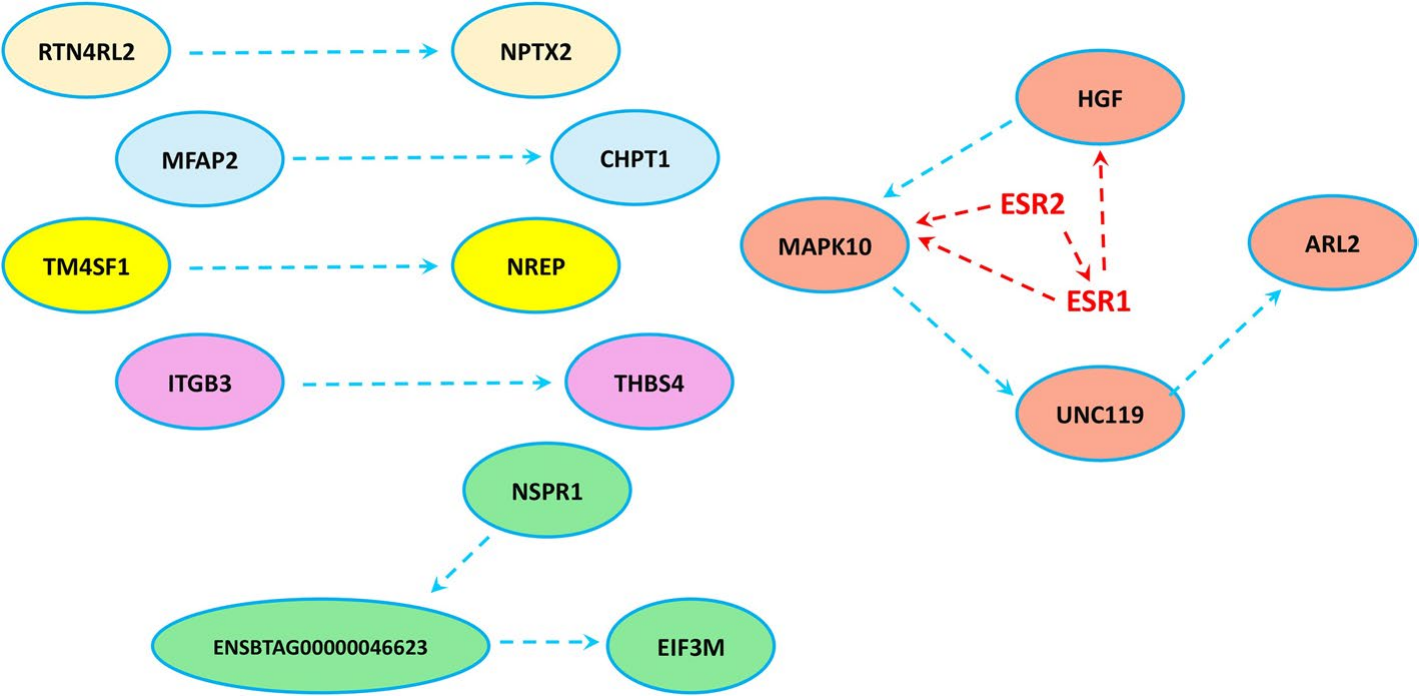
PPIs derived from STRING using upregulated genes. Interaction between ITGβ3 and THBS4 is shown. Also, MAPK10 seem to play a connective role between HGF, UNC119 and ARL2. The estrogen receptors (ESR1 and ESR2) show the hypothetic pathway of the effect of estradiol-17b in the network of the upregulated genes

**Fig. 5:**
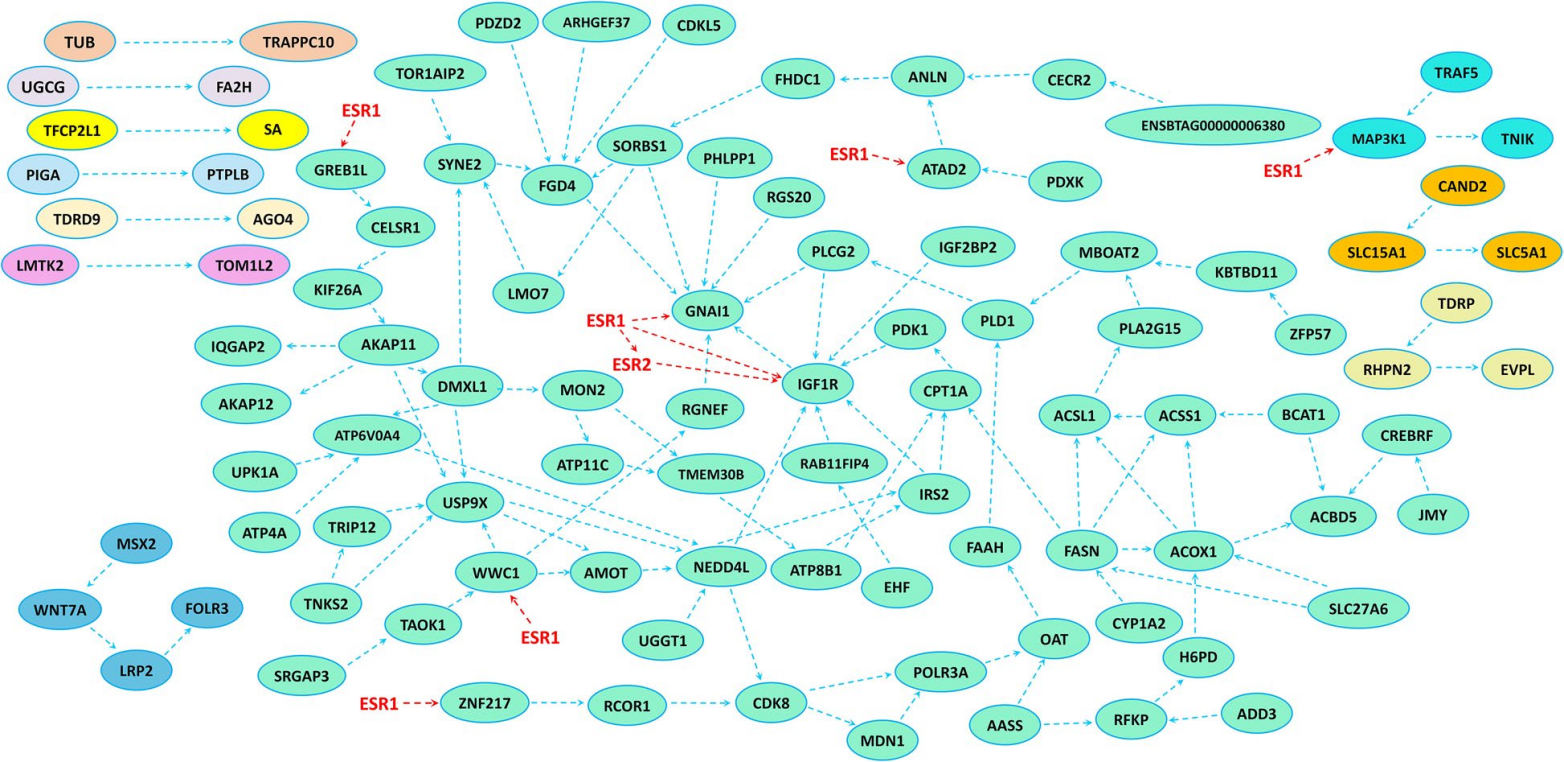
PPIs derived from STRING using downregulated genes. FAAH seems to play a connective role between CYP1A2, FASN, ACOX1, PDK1, ACSL1, PLA2G15, PLD1, OAT and AASS which are the genes involved in lipid metabolism. Also, PLCG2 and IGF1R connect lipid metabolism network to GNAI1 which seems to play a central role in the whole network with seven connections. The estrogen receptors (ESR1 and ESR2) show the hypothetic pathway of the effect of estradiol-17b in the network of the downregulated genes

Interactions between the protein of up- and down-regulated genes retrieved from STRING are shown in Figs. 4 and 5, respectively. Functional enrichment of the upregulated genes via Cytoscape did not show any significant enriched pathway. However, functional enrichment of the downregulated genes enriched lipid metabolism pathway. As shown in Fig. 4, ARL2, UCN119 and HGF are in an interacting network which links the pathway “positive regulation of cell substrate adhesion” to “peptidyl-tyrosine modification” represented in Table 2. Also, regarding the downregulated genes, the interacting network between CYP1A2, FASN, ACOX1, ACSL1, PDK1 and PLA2G15 which has been linked to AASS, OAT and PLD1 with mediatory action of FAAH is depicted (Fig. 5). These are the genes that are related to lipid metabolism as represented in Table 3. Furthermore, these two networks have been linked to GNAI1 through the mediatory action of PLCG2 and IGF1R (Fig. 5). Therefore, this may be the functional network that associates lipid metabolism to the pathway “response to platelet aggregation inhibitor”.

### Gene expression data validation using quantitative RT-PCR

**Fig. 6:**
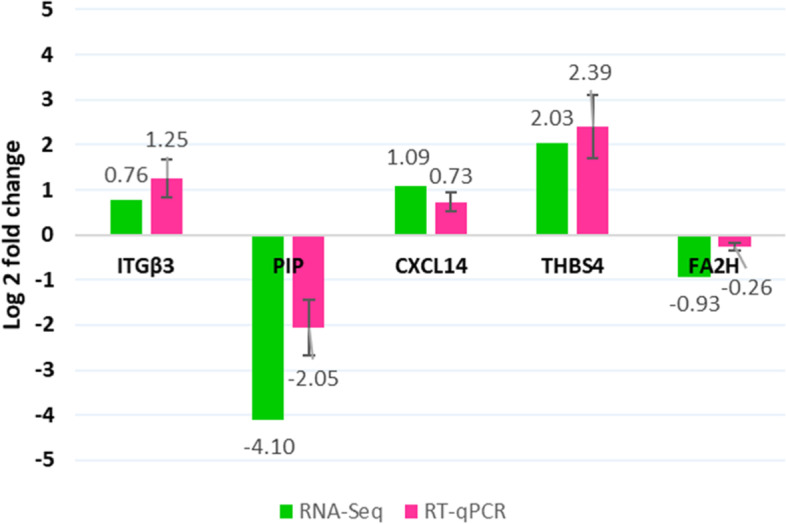
Validation of RNA-seq results by RT-qPCR. Log 2 fold change (RP to Control) obtained from each gene profiling platform (qPCR or RNA-seq) is shown by bar plots

An RT-qPCR assessment conducted to validate RNA-Sequencing (Fig. 6). Five genes that had prominent contribution to enrichment analyzes were selected for RT-qPCR. *RPS23* and *PPP2R5B* were used as reference genes [11]. The genes *ITGβ3*, *CXCL14* and *THBS4* upregulated in both RNA-seq and RT-qPCR. Also, *PIP* and *FA2H* downregulated in the both platforms. Absolute delta fold changes (∆FC; qPCR – RNA-seq) were 0.48, 0.36, 0.35, 2.04, 0.66 for the above genes, respectively. Due to similar pattern of expression, the RT-qPCR validated the results of RNA-sequencing.

## Discussion

In this study, we conducted RNA-seq on bovine placentome samples from the cows with or without RP and determined DEGs between these two groups. We also conducted a series of clinical experiments including assessment of blood parameters and histological analysis to gain more insight into the physiologic aspects of these cows.

According to the PCA plot from the RNA-seq analysis, correlation between samples showed a rather control/RP1-RP2/RP3-RP4 clustering relationship. This shows that RP1-RP2 and RP3-RP4 had a more similarity to each other. Due to the diverse etiology of RP, it is not unlikely that these samples show different clusters. However, we placed all the RP samples in one group and tried to discover the mutual enriched pathways in the RP samples that lead us to shared up- and down-regulated genes.

Over representation analysis on the up-regulated genes revealed the primary involvement of immune system in development of RP. Mature B cell differentiation and positive regulation of lymphocyte migration which were enriched by upregulated genes may occur following the liberation of the immune system from suppression around the time of parturition and activation of humoral and cellular immune responses by indirect presentation of fetal trophoblast MHC-I peptides to the lymphocytes [21, 49]. In this regard, CMTM7 and LGLAS1 promote B cell differentiation and regulate immunoglobulin production [50–52]. *ITGB3* and *CXCL14* were the enriched genes in lymphocyte migration biological process. Integrins have a key role in lymphocytes migration [53]. B lymphocytes express CXCL14 in inflammatory states and its murine homolog could stimulate chemotaxis of B cells [54, 55]. The third enriched biological process was T-helper 1 (Th1) immune response which is known to recognize MHC-I and MHC-II at the time of parturition [21, 56]. Also, this is consistent with the suggestion that there is a shift towards Th1 dominance around the time of parturition [57–59]. This dominance seems to be facilitated by IL27RA and RELB as reported previously [60, 61]. Th1 has also been associated with apoptotic events which occur in placentomes at the time of parturition and facilitates detachment of placenta [62]. Therefore, upregulation of this pathway may be due to a late effort of placentomes from RP group to detach the placenta.

Cell matrix adhesion is an important biological process that has been attributed to development of RP [2, 11]. In the present study, the enriched biological process showing cell matrix adhesion shows the importance of this process as well. *ITGβ3*, *NINJ1* and *ARL2* are the genes that have been upregulated and contributed to over-representation of this biological process. Integrins are the principal cellular components that tie the cells to their extracellular matrix. There are 18 α and 8 β subunits of integrins that constitute 24 different αβ integrin heterodimers [63–65]. ITGβ3 is a subunit β containing integrin that could contribute to making αVβ3 and αIIbβ3 integrins which are involved in cell to cell interactions and cell matrix adhesion [66, 67]. ITGβ1 has previously been detected in the cells that reside in bovine placentomes and it was suggested that this protein is involved in feto-maternal cell communications [68]. Moreover, the differences between uninucleate trophoblast cells and binuclear trophoblast giant cells regarding their interactions with their ECM environment has been attributed to the differential expression of integrins [69]. However, according to our knowledge, our result is the first report that suggests the involvement of *ITGβ3* in the etiology of RP. NINJ1 is basically a cell surface protein that mediates cell communication and could promote leukocytes migration and activity. It regulates leukocytes inflammatory response and their tissue remodeling functions in the targeted tissues. When cleaved by MMPs, this protein could reverse its functions on leukocytes [70]. ARL2 is shown to inhibit dissociation of cell adhesions in epithelial cell [71]. Therefore, its upregulation in the placentome of RP cows might also be associated with maintenance of cell attachments.

*THBS4* is also a gene that is seen frequently in the enriched pathways. PPI network in STRING showed an interaction between THBS4 and ITGβ3. THBS4 plays and important and distinct role in ECM assembly and cell-cell adhesion [72]. It regulates production, organization and assembly of collagen fibers and binds to different types of collagens and adhesive fibers on cell matrix and membranes [73]. Integrins are primary receptors for thrombospondins and integrin αvβ3 is expressed in monocytes, neutrophils and endothelial cells and acts as THBS4 receptor [74–76]. Thus, the involvement of the interaction between ITGβ3 and THBS4 and its contribution to villi-crypts attachments in placentome of the cows is plausible.

Peptidyl-tyrosine phosphorylation is also a general term which is important in cell signaling that is mainly induced by tyrosine phosphorylation [77]. The protein of the genes indicated under this enriched biological process are able to trigger signaling pathways in the cells and to induce tyrosine phosphorylation [78–81].

Initial studies on the involvement of immune system in placental expulsion showed that reduced immune function and uterine chemotaxis is a major contributor to development of RP [6, 82]. It was suggested that RP could be due to inhibited chemotaxis in cotyledons or suboptimal function of leukocytes despite the presence of chemotaxis [82]. In the present study, we showed over-representation of immune functions in RP cows that is possibly due to the more provoked inflammatory state at the placentomes region. Our results suggest that chemotactic part of immune system continues its activity in the placentomes of the cows that retain their placenta. However, its reaction may be impaired or actually delayed to detach the morphologically collapsed immature placentomes.

The first biological pathway that was enriched by the downregulated genes was glucosylceramide metabolic process. Glucosylceramide is synthesized by the UGCG enzyme [83, 84]. Glycosylated sphingolipids reside in cell membranes and are involved in cell-cell adhesion and signal transduction [85]. Glycosylated sphingolipids could further become hydroxylated by FA2H and these hFA-SLs have ranges of activities [86]. Glucosylceramides are a precursor for diverse glycosphingolipids with many cellular functions including cell differentiation, proliferation and apoptosis [87]. In epidermal cells these molecules are secreted to the intercellular membranes and contribute to normal formation of lamellar bodies in keratinocytes. Whereas silencing of FA2H decreases 2-OH glucosylceramide and interfere with formation of these lamellar bodies [88]. Whether the corporation of hydroxylated glucoceramides play a role in cell matrix adhesion, membrane structure or signal transduction of the placentome cells needs to be elucidated in future.

Most of the biological processes enriched by the downregulated genes were associated with lipid metabolic pathways. Cows are often faced with metabolic disorders during the transition period [89]. The disturbed lipid metabolism and its implication for placental detachment has been shown previously [90–93]. Thiobarbituric acid reactive substances, hidroperoxides and conjugated dienes as lipid peroxidation products were higher in placentome of cows with RP than healthy controls [90]. In another study, cows with RP were suffering from fatty liver and altered blood biochemical parameters as well [91]. High concentrations of cholesterol, NEFA, BHBA and lactate during periparturition period is associated with higher risk of development of RP [22, 92, 93]. In one study, by each 0.1 mmol/L increase in serum concentration of cholesterol or fatty acids in the week before parturition, the risk of development of RP increased by 5% [29]. Also, higher concentrations of cholesterol and NEFA were suggested as potential early predictive indicators of development of RP [93]. Our results suggest that this disturbed lipid metabolism may occur at placentomes level as well and its metabolic or hormonal implications may impair placental detachment. Reduced lipid metabolism capacity of placentomes may also make them permissive for NEFAs to impair their normal physiologic and immune functions. Several genes such as *CYP1A2*, *FASN*, *ACSL1*, *SLC27A6* and *PDK1* are seen frequently in the enriched pathways that are relating to lipid metabolism. This shows that these genes play a central role in multiple lipid metabolism oriented pathways and may make them appropriate candidate genes for further investigations.

Cytochrome P450 (CYP) is a family of enzymes which oxidize a variety of substances [94] and the expression of CYPs have previously been reported in the placentome of cattle [95, 96]. Cyp1A2 is involved in biotransformation of sex hormones specially hydroxylation of estrogen and progesterone [97–99]. In human, Cyp1A2 has been detected in placental cell line (BeWo) [100]. In the present study, the RP group showed decreased levels of *CYP1A2* compared to controls. The plasma level of E2 was also lower in RP group compared to control. Whether the lower *CYP1A2* is a cause of deficient metabolism and biotransformation of estrogens in the RP group is not clear.

FASN is involved in the synthesis of long chain fatty acids. Kusakabe et al., [101] showed that FASN is expressed in many cell types which are mainly active in lipid metabolism or hormone production and cytotrophoblasts, decidua and various fetal cells were among these FASN positive cells. This enzyme is probably responsible for *de novo* synthesis of deficient fatty acids for the placental growth or for the fetus. Therefore, its downregulation may show the disturbed metabolic events in placentomes.

The main upregulated and downregulated processes in this study were related to immune system and metabolic pathways, respectively. The similarity between the ORA and GSE analyzes also reinforces the idea that the immune system and lipid metabolism have actually become more and less active, respectively, in RP cows. Functional enrichment of downregulated genes using Cytoscape also enriched the lipid metabolic process. We may gain more insight into the etiology of RP by understanding of the links between these two systems in the body of affected cows. Some aspects of concurrent metabolic and immune imbalances in RP disorder have been described previously [21]. It has been shown that metabolic disturbances are associated with the development of RP [102]. It also was suggested that high concentrations of NEFA and cholesterol peripartum could adversely affect immune function and increase the risk of uterine diseases [24]. Metabolic stresses around the time of parturition could activate the hypothalamic-pituitary axis and increase the level of corticosteroids including cortisol [21, 103]. As a result, cortisol could suppress expression of MHC molecules and decrease prostaglandins production [21, 104]. In the present study, cortisol in RP group showed a steeper increase from day − 1 to day + 1 compared to controls and tended to be significantly different between the groups at the day of parturition. This might be associated with the observed metabolic and immunologic discrepancies that reflected in the enrichment analyzes. However, as the alternations in cortisol levels is not firmly different between the two groups, concurrent involvement of other factors in development of these discrepancies is plausible.

The concentration of E2 in the present study was higher in control group than RPs and it was significant at the day of parturition. This result is consistent with previous data [9, 35]. Progesterone level was not different between the two groups, which is consistent [35, 105] and inconsistent with previous reports [23, 36]. Our PPI analysis on up and downregulated genes showed that there are several presumptive pathways that could be affected by E2. The tendency to having higher level of cortisol in RP cows in our study is also similar to the previous studies [35, 36]. The low number of cows in our study may have prevented the CBC and hormonal assessments to become significant at some time points, but the lower concentration of E2 and higher concentration of cortisol in RP cows is according to expectations. However, our data suggests that the RP cows do not necessarily have higher P4 compared to healthy ones consistent with the data of Wischral et al., [35]. E2/P4 ratio was not significantly different between the groups. However, it was higher in control group than RP. Apart from the differences between the two groups, the time dependent changes in the hormones from days − 3 to + 1 within each group may also have effects on development of RP. For example, the level of P4 at the day − 1 was significantly higher than the day of parturition in RP groups. Also the raise in cortisol level was significantly higher at the day of parturition and day + 1 compared to day − 1 in this group. Therefore, the time point alternations in the plasma level of hormones should also be considered when concluding about the effect of hormones on development of RP.

Histologic experiment on the placentomes of the cows in this study showed that the villi-crypts interactions in RP cows remain tight and this leads to development of RP. Also maternal crypts seemed to be thicker in RP cows which may be due to the higher levels of P4 throughout the gestation or lower levels of E2. Estrogen receptors are found in stromal cells of maternal crypts during bovine pregnancy and it has been suggested that estrogens can regulate stromal cell growth in placentomes [106, 107]. Therefore, the altered paracrine activity of estrogens on stromal cells in RP cows may be a reason for thicker crypts septa in these cows.

Moretti et al., [15] have reported a significant decline in the neutrophil count between the cows with placental retention compared to healthy cows in immediate post-partum period. However, similar to our results, other hematological parameters such as lymphocytes, monocytes, hemoglobin and white blood cells were not significantly different between their experimental groups. Our data also show a tendency to reduction in the neutrophil counts immediately after parturition. However, it was not statistically significant yet on the day + 1. Altogether, the hematological parameters did not differ between the two groups. However, such as the hormonal parameters, PCV% and hemoglobin experienced a significant raise by approaching the parturition in RP and not control group, which might be due to some level of dehydration in RP cows.

In this study, we presented a productive and reproductive history for each cow and compared the means of each parameter between the RP and control groups. In this regard, mean interval between each parity throughout the cows’ life (until our last record) was not significantly different between the two groups. This could show that the cows in the both groups have had comparable past reproductive performance. In addition, the milk yield, length of gestation, age and length of last days open were not significantly different between the two groups showing that these variables may not have impacted the status of placental expulsion in the cows. However, the average parity was lower in RP group compared to controls. This was due to the limited number of calving cows (with the same parity) in the herd within a logical length of time. Although this could be a variation source in the data, prolonged sampling time to match the parities could introduce other variations (such as season effect) to the data. We presume that parity should not have an overestimated contribution to gene profiling of the retained placenta, or even if it has, our results should show the most common genes involved in retained placenta among different parities.

In a previous study, Streyl et al., [11] conducted a gene expression profiling on placentomes obtained from normal (non-RP) cows 12 days antepartum (via induced calving) and after parturition (intrapartum) using microarray platform. Physiologic processes that mainly upregulated in intrapartum group were immune response, ECM remodeling and apoptosis. In fact, their results were an anticipation of the genes which contribute to placentome maturation and normal expulsion of placenta. In the present study, we conducted RNA sequencing on placentomes extracted from RP and healthy cows within the same time after parturition and with unmanipulated calving. Therefore, our gene profiling data should provide a more equal context for comparison of RP and healthy cows and malfunctioning processes and genes involved in RP. Despite the similarities between our results with the above study, namely the enrichment of immune and cell matrix related processes, the overall processes and genes seem to have significant differences. This could originate from several issues. First, the large differences between the number of DEGs derived from the two studies. While 1226 DEGs were introduced by the previous study, the number of DEGs in the present study were 240. This could partly be a result of their higher FDR cutoff (< 0.1) besides other objective and methodology differences. Second, in the previous research, maybe not all the processes could be attributed to placental detachment due to the healthy nature of the cows investigated. In fact, some processes might be related to other mechanisms naturally occurring in preparturition period inside the placentomes. Finally, our results may show that other novel trends, not necessarily starting from weeks before parturition, could contribute to development of RP.

### Study limitations

In this study, the real-time PCR experiment to validate the RNA-seq results yielded similar up or downregulation of the five investigated genes. However, there was differences in the fold changes obtained from the two platforms (qPCR and RNA-seq). ∆FCs were < 1 for *ITGβ3*, *CXCL14* and *THBS4* and *FA2H* and = 2 for *PIP*. Deviations in differential expressions (at least in a proportion of genes) resulting from RNA-seq and qPCR is not an uncommon phenomenon and has been dealt with or occurred elsewhere [108–113]. These discrepancies could originate from various genes features such as the length and GC content, number of exons, number of paralogs and expression level or from the differences in wet lab and analysis workflows, primers and systematic quantification technologies between RNA-seq and RT-qPCR [108, 109, 112]. The source of deviations in the fold changes observed in the present study is unclear. However, due to the low ∆FCs and similar pattern of expressions, the qPCR assays are in agreement with and confirm our RNA-seq results.

Regarding the identified DEGs, a considerable issue was that a marked proportion of the genes were not characterized yet. For example, the highest upregulated (ENSBTAG00000054018) and highest downregulated (ENSBTAG00000048304) genes in RP cows did not have an identified protein. In addition, due to slower advancement in enrichment programs for analysis of RNA-seq data from Bos taurus species (compared to homo sapiens), some important pathways in the etiology of RP may remain hidden at the present time. However, the present study tried to have a wider look at the etiology of retained placenta in cattle and the genes identified in this study may be good candidates for future studies.

## Conclusion

In this study, we found 240 differentially expressed genes between the placentome of the cows with or without retained placenta. By enrichment of these genes we found that immune system pathways are over-represented and lipid metabolic pathways are under-represented in RP cows compared to controls. Genes *ITGB3*, *THBS4*, *CXCL14*, *FASN*, *FA2H*, *UGCG* and *CYP1A2* were among the genes that seem to have important role in development of RP and may prove useful candidates for future studies. We also showed that at the day of parturition E2 was significantly lower in RP cows. Therefore, our results further reinforce the role of E2 in retained placenta and show that P4 does not necessarily change in RP cows at least within the peri-parturition period. Hematological parameters were also not significantly different between the two groups, but histologic experiment showed that the villi-crypts junctions remain tighter in RP cows compared to healthy ones and this leads to retained placenta. The role of many genes identified in this study has not been previously reported in etiology of RP and our study could be used for future investigations.

## Supplementary Information

**Additional file 1:** **Table S1.** Primer sequences designed for qPCRexperiments. **Table S2.** Information about reads and alignments. **Table S3.** DEGs list. **Tables S4** and **S5** and ** Fig. S1:** GSEA analysis results.

## Data Availability

The sequencing read data as well as read counts matrix generated during this study are available in the Gene Expression Omnibus (GEO) repository under accession number GEO: GSE194033 [https://www.ncbi.nlm.nih.gov/geo/query/acc.cgi?acc=GSE194033].

## Abbreviations

RP: Retained placenta
ECM: Extracellular matrix
MMP: Matrix metalloproteinase
CPM: Count per million
E2: Estradiol-17β
P4: Progesterone
∆FC: Delta fold change
ORA: Over-representation analysis
DEGs: Differentially expressed genes
