# Supplementary material for: Gene expression profile of placentomes and clinical parameters in the cows with retained placenta
Source: BMC Genomics. 2022 Nov 21;23:760. doi: 10.1186/s12864-022-08989-5 (PMC9677913; doi:10.1186/s12864-022-08989-5)
Supplement: Supplementary file 1 — Additional file 1: Table S1. Primer sequences designed for qPCRexperiments. Table S2. Information about reads and alignments. Table S3. DEGs list. Tables S4 and S5 and Fig. S1: GSEA analysis results. [file 12864_2022_8989_MOESM1_ESM.pdf]

Supplementary information

Table S1 Primer sequences used to validate RNA-sequencing.

Target genes	Primer sequences
RPS23	F: GGTCTTCGTACTGCCAGGAA<br>R: GGCTGTTTGGCTTCAACTCC
PPP2R5B	F: TCCGCTATCAGAGCAACCAG<br>R: CTTCAGGTCAGCCACACAGT
ITGB3	F: GAAGCAGAGTGTGTCACGGA<br>R: ATGGGTCTTGGCATCAGTGG
FA2H	F: TCCATCGCTTCCTGTTCCAC<br>R: GCCGTAGCACCAGGTAGAAG
PIP	F: CCCTGCTTCTGATTCTTTGCC<br>R: GCCTTCAATCGGAATGTTGC
CXCL14	F: AAGGGACCCAAGATCCGCTA<br>R: CGTTCCAGGCGTTGTACCAT
THBS4	F: TCCTGAGGTCAGCAACCCA<br>R: CTCGTCACCAATGCCATCCTT

Table S2 Total produced reads pairs, overall alignment rate and total counted reads per sample.

	RP1	RP2	RP3	RP4	Control 1	Control 2	Control 3
**Total read pairs produced**	32921954	31286662	26533099	32367658	31181316	30012486	29412001
**Overall alignment rate**	93.66%	94.38%	94.65%	94.00%	94.51%	94.71%	94.57%
**Total counted reads aligned to known genes**	21994530	23681013	20122340	22361534	23284295	22177286	22336338

Table S3 Differentially expressed gene in RP, compared to control group.

	Gene ID	Gene symbol	Log2 fold change	FDR		Gene ID	Gene symbol	Log2 fold change	FDR
1	ENSBTAG00000054018	-	5.820607	0.002954	41	ENSBTAG00000006579	P4HA3	0.912389	0.002475
2	ENSBTAG00000013768	NPSR1	4.628598	1.60E-16	42	ENSBTAG00000013096	UNC119	0.89154	0.000854
3	ENSBTAG00000019227	-	3.591426	1.26E-06	43	ENSBTAG00000018986	ACSL4	0.87745	0.026954
4	ENSBTAG00000005585	ANXA13	3.332192	0.00626	44	ENSBTAG00000001288	MAOB	0.866447	0.02791
5	ENSBTAG00000053649	CCL5	3.251708	0.02791	45	ENSBTAG00000002490	CHPT1	0.858026	0.03201
6	ENSBTAG00000051898	FGF7	3.197084	0.032026	46	ENSBTAG00000017298	AAMDC	0.850885	0.027239
7	ENSBTAG00000013356	CATHL3	3.189129	0.001416	47	ENSBTAG00000011204	NINJ1	0.819949	0.02791
8	ENSBTAG00000045896	NPTX2	2.927327	0.003312	48	ENSBTAG00000032360	NAP1L1	0.816637	0.007293
9	ENSBTAG00000020048	MAPK10	2.8074	0.01724	49	ENSBTAG00000001721	GYG1	0.803242	0.021026
10	ENSBTAG00000045902	EPHB2	2.795297	0.03136	50	ENSBTAG00000015089	LGALS1	0.791614	0.005134
11	ENSBTAG00000012621	RTN4RL2	2.436053	0.000155	51	ENSBTAG00000002238	ARL2	0.77819	0.016521
12	ENSBTAG00000017659	BFSP2	2.345093	0.020484	52	ENSBTAG00000006209	EXT1	0.769386	0.033994
13	ENSBTAG00000045880	-	2.273439	0.003896	53	ENSBTAG00000009987	ITGB3	0.760989	0.006336
14	ENSBTAG00000039425	KRT6A	2.244221	0.004578	54	ENSBTAG00000015177	PRSS23	0.747822	0.049827
15	ENSBTAG00000050510	-	2.104372	0.001602	55	ENSBTAG00000001839	OCIAD2	0.746896	0.012294
16	ENSBTAG00000007392	SERPINA14	2.050755	9.78E-05	56	ENSBTAG00000016933	IL27RA	0.745311	0.03203
17	ENSBTAG00000012866	THBS4	2.037999	0.007293	57	ENSBTAG00000001712	CMTM7	0.73212	0.038825
18	ENSBTAG00000005104	MGAT5B	1.937658	0.021026	58	ENSBTAG00000016506	ST3GAL4	0.687857	0.031472
19	ENSBTAG00000012182	DIRAS3	1.89447	0.000643	59	ENSBTAG00000019436	SLC39A13	0.675441	0.030455
20	ENSBTAG00000013443	SDR9C7	1.783449	0.016145	60	ENSBTAG00000033252	-	0.671638	0.016179
21	ENSBTAG00000052465	-	1.612017	0.02069	61	ENSBTAG00000014695	NAA38	0.653483	0.043894
22	ENSBTAG00000006806	KRT17	1.599061	0.034069	62	ENSBTAG00000019804	SNRNP25	0.632211	0.03203
23	ENSBTAG00000006538	PTHLH	1.531825	3.36E-05	63	ENSBTAG00000000312	GRINA	0.599024	0.02791
24	ENSBTAG00000026102	PAG9	1.406669	0.03392	64	ENSBTAG00000005338	EIF3M	0.591783	0.034069
25	ENSBTAG00000037781	FKBPL	1.21142	0.03201	65	ENSBTAG00000011068	MON2	-0.59178	0.026348
26	ENSBTAG00000038428	RELB	1.185135	0.002293	66	ENSBTAG00000035230	TOR1AIP2	-0.59256	0.047064
27	ENSBTAG00000017664	HGF	1.1458	0.022177	67	ENSBTAG00000007100	TRAPPC10	-0.60266	0.025787
28	ENSBTAG00000015163	TM4SF1	1.145383	0.002705	68	ENSBTAG00000005412	NEDD4L	-0.60748	0.038629
29	ENSBTAG00000024934	-	1.125643	0.006034	69	ENSBTAG00000019181	BAIAP2L1	-0.60901	0.040019
30	ENSBTAG00000006694	CXCL14	1.09685	0.011893	70	ENSBTAG00000013221	RTTN	-0.62927	0.045509
31	ENSBTAG00000002624	PSTPIP2	1.061482	0.042932	71	ENSBTAG00000005633	ARHGEF28	-0.64794	0.026451
32	ENSBTAG00000004988	CCKAR	1.060616	0.000304	72	ENSBTAG00000015220	AGO4	-0.65526	0.03201
33	ENSBTAG00000012351	ARNT2	1.057361	0.000155	73	ENSBTAG00000003265	ADD3	-0.65733	0.031347
34	ENSBTAG00000010015	NMRAL1	1.054396	0.013269	74	ENSBTAG00000004135	LMTK2	-0.65928	0.014419
35	ENSBTAG00000011338	NREP	1.045799	0.004882	75	ENSBTAG00000015980	FASN	-0.66065	0.014419
36	ENSBTAG00000046623	-	1.035366	0.034069	76	ENSBTAG00000013880	WWC1	-0.66679	0.033711
37	ENSBTAG00000024915	SECTM1A	1.013235	0.012016	77	ENSBTAG00000005453	FGD4	-0.66792	0.024025
38	ENSBTAG00000010981	CIB2	0.990815	0.022817	78	ENSBTAG00000005444	UGGT1	-0.67094	0.037145
39	ENSBTAG00000003832	MFAP2	0.9518	0.044002	79	ENSBTAG00000016737	CDK8	-0.67139	0.024973
40	ENSBTAG00000000140	EPHX1	0.915463	0.00111	80	ENSBTAG00000011742	TNIK	-0.68061	0.045509

Table S3 continued.

	Gene ID	Gene symbol	Log2 fold change	FDR		Gene ID	Gene symbol	Log2 fold change	FDR
81	ENSBTAG00000016073	UGCG	-0.68909	0.02791	121	ENSBTAG00000033685	CCNJ	-0.80701	0.011259
82	ENSBTAG00000013790	MAP3K1	-0.69461	0.025819	122	ENSBTAG00000006242	USP9X	-0.80909	0.007331
83	ENSBTAG00000016836	PDK1	-0.6991	0.04028	123	ENSBTAG00000004879	FOXO4	-0.81014	0.039041
84	ENSBTAG00000007963	KIDINS220	-0.70131	0.025844	124	ENSBTAG00000007507	FAAH	-0.81363	0.037915
85	ENSBTAG00000002336	RCOR1	-0.70152	0.032193	125	ENSBTAG00000006748	DMXL1	-0.81572	0.008626
86	ENSBTAG00000014752	AKAP11	-0.70231	0.025844	126	ENSBTAG00000016813	SH3D19	-0.81659	0.001567
87	ENSBTAG00000023607	HACD2	-0.70549	0.03611	127	ENSBTAG00000030556	ZNF217	-0.81672	0.023391
88	ENSBTAG00000016890	ANKS1A	-0.70884	0.022817	128	ENSBTAG00000014322	FAM234B	-0.81798	0.026034
89	ENSBTAG00000017490	PLD1	-0.70906	0.014419	129	ENSBTAG00000011646	ZNF512B	-0.82007	0.024973
90	ENSBTAG00000004263	ATP6V0A4	-0.71099	0.005209	130	ENSBTAG00000002683	PFKP	-0.82164	0.016521
91	ENSBTAG00000011793	FDX1	-0.71607	0.008626	131	ENSBTAG00000021999	CPT1A	-0.82675	0.041276
92	ENSBTAG00000006928	OAT	-0.72109	0.014419	132	ENSBTAG00000004344	ACSL1	-0.83031	0.02791
93	ENSBTAG00000021653	TRIP12	-0.72183	0.010969	133	ENSBTAG00000001886	PABIR2	-0.83191	0.026279
94	ENSBTAG00000000939	KIF16B	-0.72213	0.010861	134	ENSBTAG00000003089	RHPN2	-0.83248	0.042765
95	ENSBTAG00000010693	LMO7	-0.73296	0.012135	135	ENSBTAG00000002020	CREBRF	-0.8346	0.005898
96	ENSBTAG00000045886	STARD4	-0.73604	0.032495	136	ENSBTAG00000002103	PLCG2	-0.83647	0.021237
97	ENSBTAG00000001644	MDN1	-0.7397	0.02791	137	ENSBTAG00000000897	IQGAP2	-0.83834	0.00104
98	ENSBTAG00000011322	HIPK1	-0.7416	0.012016	138	ENSBTAG00000012128	AASS	-0.84014	0.048153
99	ENSBTAG00000020561	KLHL15	-0.74527	0.008389	139	ENSBTAG00000015273	CAND2	-0.84148	0.035176
100	ENSBTAG00000031194	PHLDA2	-0.74826	0.045569	140	ENSBTAG00000015142	MAPKBP1	-0.8427	0.002896
101	ENSBTAG00000002714	GNAI1	-0.74828	0.034196	141	ENSBTAG00000009704	CECR2	-0.84313	0.008774
102	ENSBTAG00000005354	LRRFIP1	-0.74867	0.042183	142	ENSBTAG00000001825	SP6	-0.84522	0.012187
103	ENSBTAG00000017486	ADARB1	-0.75305	0.020439	143	ENSBTAG00000013873	MSX2	-0.84693	0.003093
104	ENSBTAG00000006776	KIAA0930	-0.75382	0.038825	144	ENSBTAG00000016713	TMEM30B	-0.85135	0.003032
105	ENSBTAG00000003454	RGS20	-0.77225	0.024142	145	ENSBTAG00000043957	TDRP	-0.85778	0.020504
106	ENSBTAG00000021273	SEMA4B	-0.77682	0.011104	146	ENSBTAG00000024715	PTPRJ	-0.86953	0.000192
107	ENSBTAG00000016221	AMOT	-0.77906	0.022817	147	ENSBTAG00000003983	TOM1L2	-0.87522	0.000636
108	ENSBTAG00000021026	PJA1	-0.7805	0.01838	148	ENSBTAG00000006380	-	-0.87583	0.028124
109	ENSBTAG00000002606	LGR4	-0.78083	0.004619	149	ENSBTAG00000009218	ANLN	-0.87763	0.005702
110	ENSBTAG00000038126	WIPF2	-0.78274	0.012142	150	ENSBTAG00000016484	ATP11C	-0.87798	0.002231
111	ENSBTAG00000014455	PDZD2	-0.78718	0.017263	151	ENSBTAG00000010999	IL17RD	-0.89491	0.00626
112	ENSBTAG00000010350	USP53	-0.78757	0.034069	152	ENSBTAG00000004746	HELZ	-0.89623	0.000478
113	ENSBTAG00000005738	ATP8B1	-0.79324	0.009415	153	ENSBTAG00000019781	CLASP1	-0.90282	0.008675
114	ENSBTAG00000002880	SORBS1	-0.79408	0.028747	154	ENSBTAG00000049368	-	-0.9113	0.03201
115	ENSBTAG00000007646	PIGA	-0.79414	0.028124	155	ENSBTAG00000025856	JMY	-0.91641	0.037108
116	ENSBTAG00000016529	SLC25A30	-0.79815	0.000486	156	ENSBTAG00000001186	SH2D4A	-0.91667	6.01E-05
117	ENSBTAG00000025450	SYNE2	-0.80095	0.026954	157	ENSBTAG00000004281	ACSS1	-0.91893	0.006704
118	ENSBTAG00000017278	ACBD5	-0.80477	0.003013	158	ENSBTAG00000003101	SALL4	-0.91915	0.006704
119	ENSBTAG00000002002	ATAD2	-0.80541	0.001673	159	ENSBTAG00000021904	KIF26A	-0.92808	0.002526
120	ENSBTAG00000016991	EFNB2	-0.80631	0.022177	160	ENSBTAG00000051782	ATAD2	-0.9302	0.038268

Table S3 continued.

	Gene ID	Gene symbol	Log2 fold change	FDR		Gene ID	Gene symbol	Log2 fold change	FDR
161	ENSBTAG00000007765	TFCP2L1	-0.93154	0.008774	201	ENSBTAG00000047939	ZNF75D	-1.24068	0.007331
162	ENSBTAG00000012222	FA2H	-0.93228	0.000854	202	ENSBTAG00000010517	EVPL	-1.29617	0.01838
163	ENSBTAG00000007512	PLA2G15	-0.93769	0.00495	203	ENSBTAG00000008036	CELSR1	-1.29617	4.63E-07
164	ENSBTAG00000017836	ZFP57	-0.94913	0.000854	204	ENSBTAG00000045734	LRRC26	-1.37672	0.012016
165	ENSBTAG00000043963	FHDC1	-0.94985	0.006215	205	ENSBTAG00000014788	AKAP12	-1.38366	6.14E-07
166	ENSBTAG00000000827	TAOK1	-0.96068	0.004578	206	ENSBTAG00000004662	SLC16A12	-1.39291	0.033994
167	ENSBTAG00000016972	RAB11FIP4	-0.96252	0.000259	207	ENSBTAG00000048529	-	-1.40667	0.026756
168	ENSBTAG00000013825	BCAT1	-0.9697	0.000732	208	ENSBTAG00000012020	TRAF5	-1.43909	0.028124
169	ENSBTAG00000012019	IRS2	-0.97018	0.031882	209	ENSBTAG00000004685	SRGAP3	-1.44364	0.003794
170	ENSBTAG00000054250	-	-0.97802	0.009481	210	ENSBTAG00000052132	FOXQ1	-1.4618	0.014515
171	ENSBTAG00000009314	FYB2	-0.98033	0.003914	211	ENSBTAG00000002982	PITPNM3	-1.50605	0.022354
172	ENSBTAG00000001668	WNT7A	-0.98209	0.000863	212	ENSBTAG00000017882	LRRC3	-1.51903	0.003013
173	ENSBTAG00000001405	GLCE	-0.98413	2.16E-05	213	ENSBTAG00000047426	LYPD6	-1.5244	0.039271
174	ENSBTAG00000030671	HOOK1	-0.99516	0.000125	214	ENSBTAG00000018284	KBTBD11	-1.53812	0.032222
175	ENSBTAG00000013204	ATP13A3	-0.99765	0.000255	215	ENSBTAG00000048086	-	-1.5662	0.001649
176	ENSBTAG00000008160	MBOAT2	-0.99933	7.90E-05	216	ENSBTAG00000004862	TUB	-1.56936	0.007293
177	ENSBTAG00000014615	SLC26A2	-1.01027	0.002475	217	ENSBTAG00000020543	UPK1A	-1.57437	0.039139
178	ENSBTAG00000034560	-	-1.01843	0.02791	218	ENSBTAG00000011634	ACP3	-1.57457	1.88E-05
179	ENSBTAG00000018186	PDXK	-1.02511	0.001325	219	ENSBTAG00000022837	-	-1.58341	0.030455
180	ENSBTAG00000015419	ARHGEF37	-1.02818	0.000192	220	ENSBTAG00000000920	DNAH17	-1.60956	0.016034
181	ENSBTAG00000001754	AHCYL2	-1.03185	0.04028	221	ENSBTAG00000006907	NEB	-1.63022	0.003129
182	ENSBTAG00000013259	POLR3A	-1.03548	0.001634	222	ENSBTAG00000048540	-	-1.73164	0.033496
183	ENSBTAG00000030174	ACOX1	-1.04647	0.007248	223	ENSBTAG00000049220	-	-1.79718	0.000287
184	ENSBTAG00000004440	SLC15A1	-1.04767	0.016034	224	ENSBTAG00000054268	-	-1.86422	0.02791
185	ENSBTAG00000015802	CREB3L2	-1.04982	0.02791	225	ENSBTAG00000038532	FOLR3	-1.88011	0.005209
186	ENSBTAG00000045832	PHLPP1	-1.05873	0.001325	226	ENSBTAG00000013736	PROM1	-1.91426	0.005945
187	ENSBTAG00000009279	TNKS2	-1.06297	0.000546	227	ENSBTAG00000015915	SBSN	-2.01054	0.001351
188	ENSBTAG00000006013	NT5DC3	-1.06998	0.000972	228	ENSBTAG00000020269	MISP	-2.01946	0.000255
189	ENSBTAG00000004555	LRP2	-1.08673	0.000102	229	ENSBTAG00000004860	SLC27A6	-2.13222	0.045569
190	ENSBTAG00000022733	EPM2AIP1	-1.09191	0.010302	230	ENSBTAG00000012851	SLC5A1	-2.31515	0.02791
191	ENSBTAG00000023429	PLS1	-1.09875	0.000594	231	ENSBTAG00000021425	LMAN1L	-2.32427	0.013934
192	ENSBTAG00000021527	IGF1R	-1.11261	0.001325	232	ENSBTAG00000016514	CPE	-2.5164	0.026954
193	ENSBTAG00000019229	TMEM144	-1.16185	0.000259	233	ENSBTAG00000008777	ATP4A	-2.66466	0.005455
194	ENSBTAG00000023407	B3GALT5	-1.16277	0.00402	234	ENSBTAG00000020402	TDRD9	-2.83899	7.73E-08
195	ENSBTAG00000017150	EHF	-1.18396	0.003825	235	ENSBTAG00000000085	CYP1A2	-2.86827	0.042603
196	ENSBTAG00000008275	GREB1L	-1.18685	1.88E-05	236	ENSBTAG00000054413	-	-3.64553	0.021026
197	ENSBTAG00000007666	IGF2BP2	-1.19286	1.43E-07	237	ENSBTAG00000002576	GLDN	-3.80061	8.12E-07
198	ENSBTAG00000004246	H6PD	-1.19723	1.63E-05	238	ENSBTAG00000006655	PIP	-4.10712	2.02E-15
199	ENSBTAG00000009337	PNMA8A	-1.19844	0.029025	239	ENSBTAG00000023563	-	-4.21423	0.000148
200	ENSBTAG00000007428	CDKL5	-1.22504	3.24E-07	240	ENSBTAG00000048304	-	-5.55843	2.93E-13

Table S4 Upregulated biological processes enriched by GSEA and their corresponding genes.

**Upregulated biological processes enriched by GSEA and their associated genes**
**Leukocyte migration**
CXCR3, XCL2, CCL8, MMP9, THBS4, CCL22, CD74, IL1B, TBX21, CXCL2, CXCL8, ITGB7, CCL25, TNF, S100A9, CCR7, LOC529196, SELL, CHGA, CCL2, CORO1A, CXCL14, ITGB2, RARRES2, C3AR1, JAML, S100A8, PYCARD, GRO1, AIF1, CCL1, CXCR1, CCR2, SPN, FCER1G, ITGB3, CCL28, THY1, CX3CR1, TRPM2, CXCR2, CCR1, LGALS3, MCOLN2, IL1RN, PTGER4, CCL17, EDNRB, SYK, C5AR1, MIF, EMILIN1, CCL3, RAC2, AKIRIN1, SELP, RPS19, SLAMF1, ARHGEF5, FADD, ANXA1
**Innate immune response**
SH2D1A, CRTAM, CATHL1, CAMP, XCL2, GBP5, CCL8, IL18, CCL22, LEP, COCH, KYNU, GFI1, SLPI, CCL25, S100A9, C3, CHGA, CCL2, CORO1A, CATHL5, C1QB, C2, IRF1, ISG15, TLR2, CLEC7A, C1S, C1QA, S100A12, RARRES2, NLRP3, SNCA, S100A8, PYCARD, MX2, AIF1, CCL1, TMEM173, VNN1, IRF8, FCER1G, TSPAN6, COLEC12, SAMHD1, CLEC5A, KRT1, JCHAIN, TLR7, TNFAIP8L2, NCR3, IFI6, CCL17, RSAD2, LBP, IRF5 IFITM3, SYK, UBE2K, CASP4, FGR, NLRC4, CFB, PIK3R6, IFITM2, BPIFB1, MIF, IFIH1, MX1, FCN1, IL34, MR1, CCL3, RIPK2, C7H19orf66, LTF, DUSP10, STX8, LY96, RPS19, TRIM38, PARP9, LOC100850276, FADD, CD14, ZYX, ANXA1
**Adaptive immune response**
SH2D1A, CD8A, IL4, IL18, CD274, CD74, IL1B, TBX21, LOC515418, ITK, TNF, C3, B2M, GAPT, RELB, C1QB, SASH3, C2, C1R, C1S, C1QA, BATF, NLRP3, CTSS, PRKCQ, BTK, IL7R, CTSH, CCR2, FYN, FCER1G, IL27RA, BCL3, PRKCB, JCHAIN, ERAP2, SAMSN1, FCGR1A, BoLA, RSAD2, SYK, ERCC1, SEMA4A, RIPK2, DUSP10, LTA, SLAMF1, LOC100850276 ,FADD, ANXA1, HLX
**Antigen processing and presentation**
BOLA-DQA5, CD74, BOLA-DRA, BOLA-DMB, LOC515418, PSMB8, CCR7, TAP1, B2M, RELB, CLEC4A, CTSS, PYCARD, TREM2, NOD1, IFI30, FCER1G, ERAP2, FCGR1A, BoLA, RAB32, LOC618733, MR1
**Cell activation**
PLA2G5, SIT1, CD8A, IL4, EOMES, IL18, CD274, FCGR3A, CD74, CD5, TBX21, LEP, LCK, CXCL8, IL1RL2, ICOS, CDH17, CD244, ITK, CD40, CCR7, CD38, CHGA, B2M, PSMB10, GAPT, CORO1A, CSF2, RELB, SASH3, IL10, IRF1, TLR2, CLEC4A, ITGB2, BATF, PIK3CG, PLEK, CCR9, PLA2G3, S100A12, JAML, NLRP3, PRKCQ, SNCA, PYCARD, BTK, IL7R, AIF1, LDLR, MEGF10, CARD11, BATF2, S100A13, IKZF1, CCR2, NR4A3, FERMT3, IHH, TNFSF9, SPN, FYN, BCL11B, VNN1, LGALS1, ZAP70, FCER1G, CD86, SPI1, ITGB3, MAFB, RAB29, IL27RA, BCL3, THY1, CMTM7, LFNG, PRKCB, CDK6, SAMSN1, APBB1IP, TNFAIP8L2, EGR3, CD40LG, NCR3, MMP14, ENPP3, SLC46A2, LMO4, LGALS3, CHRNB2, PTGER4, RSAD2, LBP, RASAL3, SYK, FKBP1B, LCP1, FGR, DCSTAMP, PIK3R6, MIF, EMILIN1, ERCC1, SEMA4A, CCL3, BATF3, RAC2, BCL2, TGFBR1, DUSP10, RHOH, RPL22, SLAMF1, FOXN1, LOC100850276, PDGFRA, FADD, ANXA1, ADORA2B, HLX, SOCS1, RPS3, TRAF6, NLRC3
**Humoral immune response**
SH2D1A, CATHL1, CAMP, CXCL2, CXCL8, SLPI, TNF, S100A9, CCR7, C3, CATHL5, C1QB, C2, C1R, C1S, C1QA, RARRES2, GRO1, CCR2, BCL3, KRT1, JCHAIN, CFB, FCN1, LTF, RPS19, LTA
**Taxis**
CXCR3, XCL2, CCL8, THBS4, CCL22, CD74, IL1B, CXCL2, CXCL8, PAX6, CCR5, CCL25, S100A9, CCR7, CXCR4, LOC529196, CRMP1, CHGA, CCL2, CORO1A, HGF, CXCL14, ITGB2, CCR9, RARRES2, C3AR1, ETV1, LHX3, JAML, PRKCQ, S100A8, SEMA3B, GRO1, NTF3, PTAFR, AIF1, CUBN, CCL1, CXCR1, CDK5R1, CCR2, NR4A3, LPAR1, LSP1, BCL11B, FCER1G, EXT1, CCL28, L1CAM, CX3CR1, TRPM2, EGR3, CXCR2, CCR1, RPL24, NCAM1, LGALS3, IL1RN, EPHB3, CCL17, EDNRB, SYK, C5AR1, MIF, CSF1R, PLXNC1, SEMA4A, RAB13, RHOG, CCL3, RAC2, AKIRIN1, RPS19, SLAMF1, ARHGEF5, PDGFRA, ANXA1, CCRL2
**Positive regulation of immune system process**
SH2D1A, CRTAM, XCL2, IL4, GBP5, IL18, CD274, THBS4, CD74, CD5, IL1B, TBX21, LEP, LCK, LIF, COCH, IL1RL2, ICOS, GFI1, LOC515418, CD244, ITK, TNF, CCR7, LOC529196, C3, CD38, B2M, CORO1A, EVI2B, C1QB, SASH3, C2, IRF1, ISG15, TLR2, C1R, CXCL14, C1S, C1QA, ITGB2, RARRES2, C3AR1, NLRP3, PRKCQ, PYCARD, RUNX1, BTK, IL7R, AIF1, CARD11, TREM2, CCR2, NR4A3, TMEM173, NOD1, IHH, TNFSF9, FYN, VNN1, LGALS1, ZAP70, FCER1G, CD86, FYB1, ITGB3, TSPAN6, RAB29, IL27RA, COLEC12, THY1, PRKCB, KRT1, BLK, TLR7, EGR3, CD40LG, NCR3, MMP14, CCR1, ENPP3, FCGR1A, AQP3, LGALS3, KCNN4, BoLA, CHRNB2, RSAD2, RASAL3, SYK, UBE2K, FGR, NLRC4, C5AR1, DCSTAMP, CFB, PIK3R6, MIF, IFIH1, LOC525599, FCN1,IL34, CCL3, RIPK2, RAC2, BCL2, LTF, DUSP10, AKIRIN1, LY96, RPS19, LTA, SLAMF1, PARP9, LOC100850276, FADD, CD14, ANXA1, ADORA2B, HLX, SOCS1, RPS3, TRAF6
**Immune effector process**
SH2D1A, CRTAM, CD8A, IL4, EOMES, IL18, CD74, IL1B, TBX21, LEP, CDH17, LOC515418, CD244, TNF, C3, CHGA, B2M, GAPT, CORO1A, RELB, C1QB, SASH3, C2, IL10, IRF1, ISG15, C1R, C1S, C1QA, ITGB2, BATF, PLA2G3, S100A12, NLRP3, PYCARD, BTK, MX2, IL7R, CTSH, S100A13, CCR2, NR4A3, TMEM173, LGALS1, FCER1G, TSPAN6, IL27RA, BCL3, SAMHD1, LFNG, KRT1, TLR7, APBB1IP, NCR3, ENPP3, FCGR1A, MAP3K14, BoLA, PTGER4, IFI6, RSAD2, LBP, IRF5, IFITM3, IFI44L, SYK, LCP1, FGR, CFB, PIK3R6, IFITM2, MIF, IFIH1, MX1, FCN1, ERCC1, SEMA4A, CCL3, RAC2, BCL2, C7H19orf66, DUSP10, RPS19, LTA, SLAMF1, TRIM38, PARP9, LOC100850276, FADD, ANXA1, ADORA2B, HLX
**Cytokine metabolic process**
IL18, IL1A, TNF, LTB, IL10, IRF1, PRKCQ, CARD11, CCR2, NOD1, GHSR, BCL3, CX3CR1, TLR7, SYK

Bolded biological processes shows that they were enriched by FDR < 0.05.

Table S5 Downregulated biological processes enriched by GSEA and their corresponding genes.

**Downregulated biological processes enriched by GSEA and their associated genes**
Dicarboxylic acid metabolic process
HAL, GGT1, DDO, GPT2, QPRT, PM20D2, SLC1A3, OAT, TAT, GGT5, ASS1, ADHFE1, IDH1, NMNAT2, GLUD1, GCLM, MTRR, KYAT3, HAAO, ASL, AMDHD1, GOT1L1, OGDH, MDH1, GCLC
Isoprenoid metabolic process
CYP26C1, CYP1A2, CYP26B1, CYP26A1, ALDH8A1, RDH12, CRABP1, DHRS9, DHRS3
Demethylation
CYP1A2, USP9X, GATA3, APOBEC2, KDM2B
Regulation of cyclase activity
NOS2, ORAI1, FSHR, PDZD3, STIM1
Sulfur compound metabolic process
MAT1A, ACPP, GSTA1, CHST6, SULT4A1, GGT1, GLCE, ELOVL5, AASS, PDK1, DLD, PTGES, GGT5, CDO1, PDHA1, CBS, GNS, CHST3, ACACA, ELOVL7, ADI1, IDH1, HYAL4, CHST8, FAR1, LPO, GPAM, PDHX, CTNS, MMUT, CIAO1, SULT1B1, ENPP1, MICAL1, GCLM, MTRR, OPLAH, MLYCD, SLC35D1
Regulation of lyase activity
NOS2, ORAI1, FSHR, PDZD3, STIM1
Small molecule catabolic process
CYP26C1, CYP26B1, MAT1A, PAH, HAL, CYP26A1, NOS2, ACOX1, LDHD, ACADS, LIPE, DDO, AASS, QPRT, PM20D2, ABCD3, OAT, TAT, PADI4, SESN2, SORD, CRABP1, GALM, PPARD, CPT1B, URAD, PDE2A, ADHFE1, APOBEC2
pri-miRNA transcription by RNA polymerase II
SMAD1, PPARD, NFATC3, GATA2, SMAD4, SMAD6, TGFB2, APLN, NGFR, ATOH8, BMPR1A
Response to xenobiotic stimulus
CYP1A2, CYP26B1, ABCB4, CYP26A1, UGT1A1, N6AMT1
Lipid catabolic process
CYP26C1, CYP1A2, CYP26B1, PNPLA1, CYP26A1, ACOX1, ACADS, LIPE, PLA2G15, GM2A, GDPD3, ABCD3, SMPDL3B, PLBD1, PLD1, GLA, SESN2, ABHD5, PLCZ1, CRABP1, PPARD, CPT1B, PNPLA2, PLD3, APOA1, ABHD4, ABHD6, PLCD3, IDH1, PRKCE, HSD17B11, ECHDC2, PLA2G7

The enriched processes are not bolded showing their FDR > 0.05.

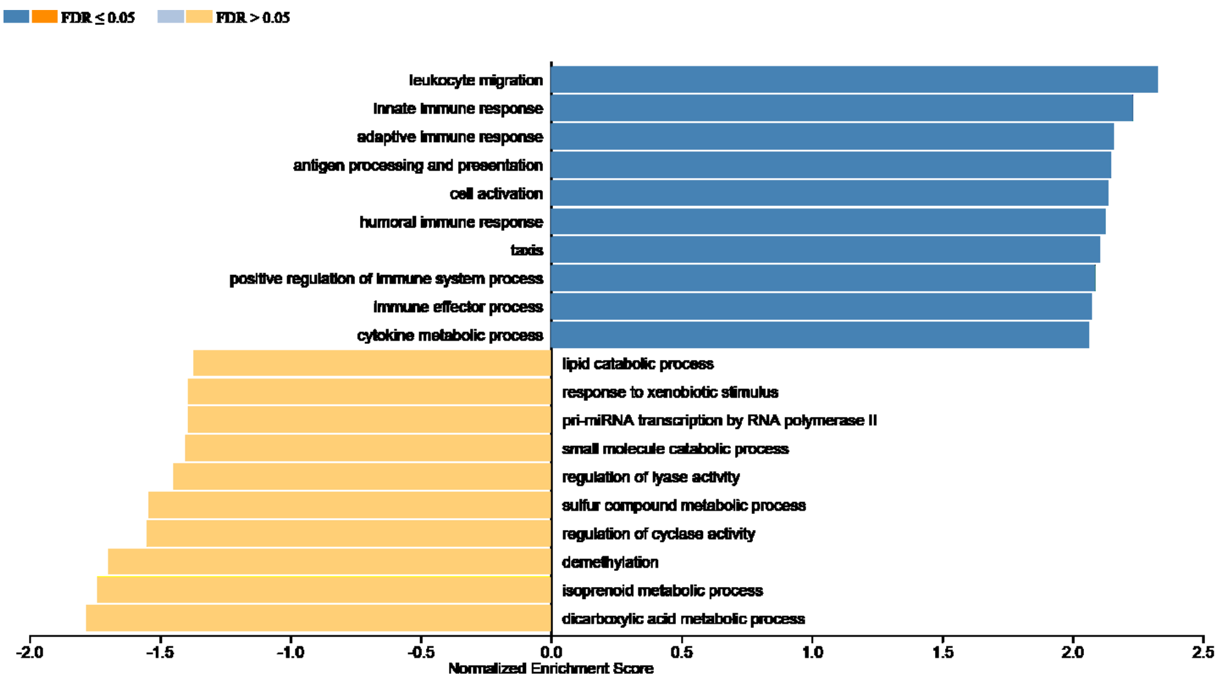

Fig. S1 Gene set enrichment analysis using all analyzed genes in this study. As indicated, all upregulated biological processes are related to immune system. On the other hand, most of the enriched pathways by downregulated genes are related to metabolic processes. Lipid metabolism seems to be the main pathway that is downregulated.
